# Supplementary material for: Towards higher frequencies in a compact prebunched waveguide THz-FEL
Source: Nat Commun. 2024 Aug 31;15:7582. doi: 10.1038/s41467-024-51892-8 (PMC11365952; doi:10.1038/s41467-024-51892-8)
Supplement: Supplementary file 1 — Supplementary Information [file 41467_2024_51892_MOESM1_ESM.pdf]

Supplementary Information for "Towards higher frequencies in a compact prebunched waveguide THz-FEL"

A. Fisher, M. Lenz, A. Ody, Y. Yang, and P. Musumeci
UCLA Department of Physics and Astronomy

C. Pennington and J. Maxson
Cornell University, Ithaca, NY

T. Hodgetts, R. Agustsson, and A. Murokh
Radiabeam Technologies

(Dated: August 1, 2024)

SUPPLEMENTARY NOTE 1: PREBUNCHING

The UV laser pulse is shaped with a set of birefringent α-BBO crystals with discrete thicknesses to create an electron beam consisting of multiple beamlets. It is not immediately obvious which specific crystals will maximize the FEL emission in the undulator since, off the zero slippage resonance, this depends both on the beam bunching factor and the temporal structure of the current density in the undulator.

To motivate the choice for the experiment, Figure 1 compares the transport and bunching spectra for electron beams created using 8,4,2 mm or 8,4,1 mm crystals. A non-uniform beamlet spacing allows space charge to induce energy chirp within the beamlets, improving the longitudinal overlap after compression. The histogram projections show that the red beamlets overlap to create three density peaks while the blue beamlets remain distinct with a lower peak current and irregular spacing. Reducing the longitudinal compression to increase the separation of the blue beamlets would significantly reduce the beam current density.

We note that for different beamlines the particular choice of alpha-BBO thicknesses employed might need to be modified, since the longitudinal phase space evolution is impacted by many factors including the RF gun and linac phases and accelerating gradients, the transverse envelope along the beamline (which at UCLA Pegasus is predetermined by the existing aperture such as the 8 mm linac irises), the R56 of the chicane, and above all the target resonant frequency.

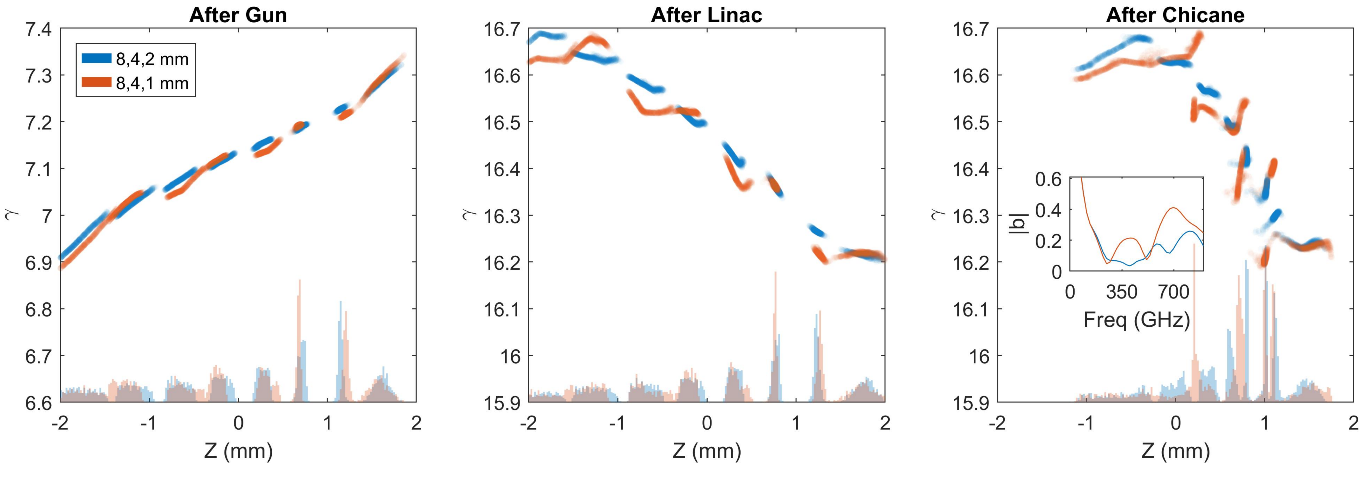

FIG. 1. Evolution of longitudinal phase space for two different choices of α-BBO crystal sets.

SUPPLEMENTARY NOTE 2: THZ STABILITY

Since the beam structure is generated at the cathode by suitably shaping the photocathode driver laser, the THz waveform is phase-locked to the optical laser pulse. The largest variations in shot-to-shot EOS signal are observed in the relative time-of-arrival at the ZnTe crystal which translates into a horizontal jitter in the EOS image structure.

Note that this timing jitter is the main reason for the implementation of a single-shot EOS diagnostic as opposed to one where the relative delay between the IR laser and the THz radiation is scanned over many shots via a delay stage.

In order to correct for this time-of-arrival jitter, we perform a simple realignment of the waveforms by maximizing the overlap between the peaks in post-processing of the data. This provides us with a direct measurement of the rms time-of-arrival jitter yielding 1 ps. In this process, the outliers in the time-of-arrival distribution greater than 3 ps from the mean (less than 10% of the total shots) are removed from the data. Not surprisingly, the time traces for the outlier shots have the largest differences from the average waveform, due to the variation of the RF phases and ensuing compression dynamics experienced by the beam.

Fig. 2 shows measurements of the high frequency radiation pulse for a beam energy of $\gamma = 16.3$ where timing jitter has been removed and shots are ordered by time of arrival. The signal amplitudes vary consistently with the fluctuations in bunch charge which is measured to have 6 pC rms jitter about the nominal 250 pC value, but the temporal structure remains consistent shot to shot. This is to be expected since the radiation signal in our FEL system does not grow from noise, but is seeded with a well defined, prebunched structure.

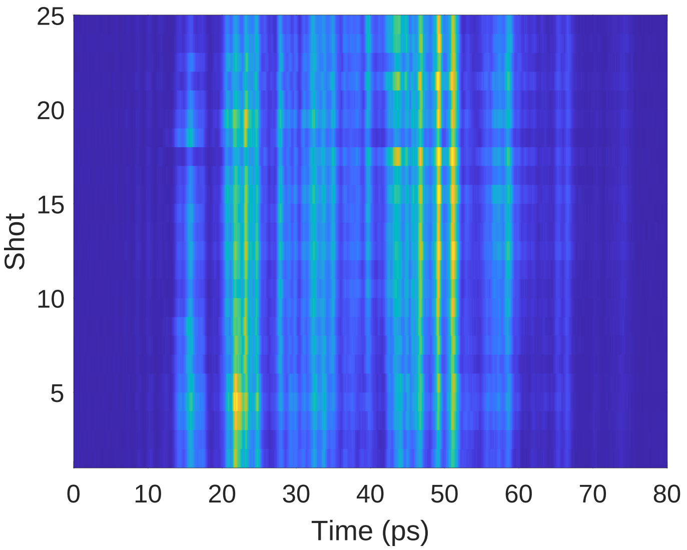

FIG. 2. Shot to shot consistency of EOS measurements of high frequency content for a beam energy of $\gamma = 16.3$. Shots are ordered by time of arrival.

SUPPLEMENTARY NOTE 3: HIGH FREQUENCY SIMULATION

In order to demonstrate the possibility to extend the scheme discussed in the paper to higher frequencies, we present here a simulation study for a scaled version of our experiment where the zero-slippage resonance is increased to 0.4 THz and we target resonance at 1.5 THz with a beam energy of $\gamma = 23.2$ by artificially increasing the linac accelerating gradient to 32 MV/m. The undulator parameters are unchanged and the waveguide radius is reduced to 1.42 mm.

A bunching factor of 0.33 can be achieved using the same set of 8,4,1 mm α-BBO crystals with similar beam transport and optimized gun phase (26°) and linac phase (17°). The injected longitudinal phase space is shown in Fig. 3 with the corresponding current profile and associated bunching factor. Given a nearly ideal transmission of 250 pC through the undulator, self-consistent GPT-FEL simulations indicate that a pulse energy of 28 μJ is produced at and above 1.5 THz (spectrum shown in the inset of Fig. 3b). The output longitudinal phase space shows clear evidence of the complex longitudinal dynamics resulting from the interaction of the THz radiation emitted by the beam with the electrons in the undulator. Of course, the smaller radius increases constraints on undulator tuning and waveguide alignment such that significant charge transmission might be difficult to achieve in practice.

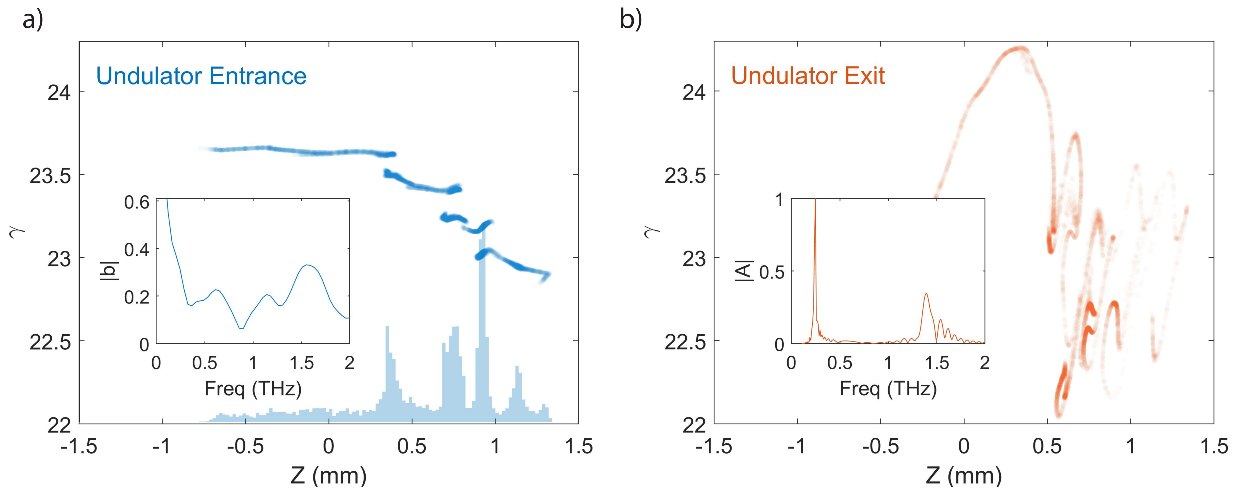

FIG. 3. GPT simulation targeting resonance at 1.5 THz. a) The longitudinal phase space is shown at the undulator entrance with histogram projection. A bunching factor of 0.33 is achieved using 8,4,1 mm α-BBO crystals. b) The decelerated beam shows clear pondomotive buckets and the spectrum shows the expected high (28 μJ) and low (21 μJ) resonant frequencies.

SUPPLEMENTARY NOTE 4: CHICANE FOCUSING

A compact, permanent magnet chicane (180 x 60 x 300 mm^3) with an R56 of 6.4 cm was installed on the beamline to compress the beam within a short distance. The outer and inner chicane magnets are offset -13 mm and -50 mm in $\hat{x}$ relative to the beam axis to target an energy acceptance of $15 < \gamma < 17$, and the pole angles ($\theta_1 = 105°$, $\theta_2 = 100°$) are chosen to minimize dispersion and equally distribute focusing in the transverse dimensions. Chicane focusing was measured with raster scans of a steering magnet 26 cm upstream of the chicane entrance, imparting a 5 mrad/A kick with beam position measured on two screens, 15 cm and 83 cm downstream of the chicane exit.

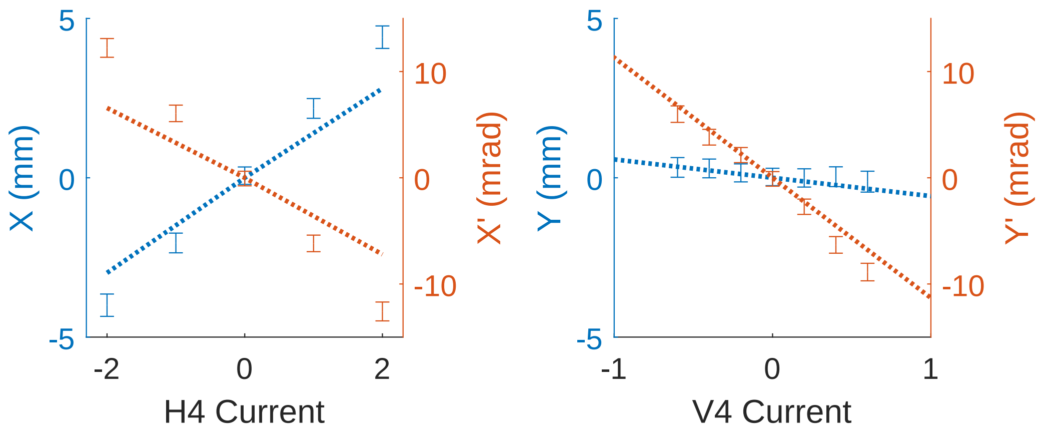

FIG. 4. Exit position and angle measurements as a function of upstream steering against modified RADIA model (dotted lines). Data inferred from two screens 15 cm and 83 cm downstream with error bars showing the standard deviation of 30 shots.
